# RETRACTION: Effectiveness of Prophylactic Application of Negative Pressure Wound Therapy in Stopping Surgical Site Wound Problems for Closed Incisions in Breast Cancer Surgery: A Meta‐Analysis

**DOI:** 10.1111/iwj.70459

**Published:** 2025-04-02

## Abstract

**RETRACTION:** SongJ. , LiuX. , and WuT. , “Effectiveness of Prophylactic Application of Negative Pressure Wound Therapy in Stopping Surgical Site Wound Problems for Closed Incisions in Breast Cancer Surgery: A Meta‐Analysis,” International Wound Journal 20, no. 2 (2023): 241–250, 10.1111/iwj.13866.35726346 PMC9885480

The above article, published online on 20 June 2022, in Wiley Online Library (http://onlinelibrary.wiley.com/), has been retracted by agreement between the journal Editor in Chief, Professor Keith Harding; and John Wiley & Sons Ltd. Following an investigation by the publisher, all parties have concluded that this article was accepted solely on the basis of a compromised peer review process. The editors have therefore decided to retract the article. The authors did not respond to our notice regarding the retraction.

**RETRACTION:** J. Song , X. Liu , and T. Wu , “Effectiveness of Prophylactic Application of Negative Pressure Wound Therapy in Stopping Surgical Site Wound Problems for Closed Incisions in Breast Cancer Surgery: A Meta‐Analysis,” International Wound Journal 20, no. 2 (2023): 241–250, 10.1111/iwj.13866.35726346 PMC9885480

The above article, published online on 20 June 2022, in Wiley Online Library (http://onlinelibrary.wiley.com/), has been retracted by agreement between the journal Editor in Chief, Professor Keith Harding; and John Wiley & Sons Ltd. Following an investigation by the publisher, all parties have concluded that this article was accepted solely on the basis of a compromised peer review process. The editors have therefore decided to retract the article. The authors did not respond to our notice regarding the retraction.

